# Per- and Polyfluoroalkyl Substances (PFAS) Affect Female Reproductive Health: Epidemiological Evidence and Underlying Mechanisms

**DOI:** 10.3390/toxics12090678

**Published:** 2024-09-18

**Authors:** Rui Qu, Jingxuan Wang, Xiaojie Li, Yan Zhang, Tailang Yin, Pan Yang

**Affiliations:** 1 Reproductive Medicine Center, Renmin Hospital of Wuhan University, Wuhan 430060, China; qr200111@163.com (R.Q.); w009988776@163.com (J.W.); 2 Department of Public Health and Preventive Medicine, School of Medicine, Jinan University, Guangzhou 510632, China; lixiaojie101121@163.com (X.L.); yangpan@jnu.edu.cn (P.Y.); 3 Department of Clinical Laboratory, Renmin Hospital of Wuhan University, Wuhan 430060, China; 4 China Greater Bay Area Research Center of Environmental Health, School of Medicine, Jinan University, Guangzhou 510632, China; 5 Key Laboratory of Viral Pathogenesis & Infection Prevention and Control, Jinan University, Ministry of Education, Guangzhou, 510632, China

**Keywords:** PFAS, female reproduction, reproductive endocrine disorders, pregnancy complication, adverse pregnancy outcome

## Abstract

PFAS (per- and polyfluoroalkyl substances) have been extensively used across numerous industries and consumer goods. Due to their high persistence and mobility, they are ubiquitous in the environment. Exposure to PFAS occurs in people via multiple pathways such as dermal contact, water supply, air inhalation, and dietary intake. Even if some PFAS are being phased out because of their persistent presence in the environment and harmful impacts on human health, mixes of replacement and legacy PFAS will continue to pollute the ecosystem. Numerous toxicological investigations have revealed harmful effects of PFAS exposure on female reproductive health, e.g., polycystic ovaries syndrome, premature ovarian failure, endometriosis, reproductive system tumors, pregnancy complications, and adverse pregnancy outcomes. Despite extensive epidemiological studies on the reproductive toxicity of PFAS, research findings remain inconsistent, and the underlying mechanisms are not well understood. In this review, we give an in-depth description of the sources and pathways of PFAS, and then review the reproductive toxicity of PFAS and its possible mechanisms.

## 1. Introduction

Per- and polyfluoroalkyl substances (PFAS) are a class of hydrocarbons where hydrogen atoms are fully or partially replaced by fluorine atoms. Presently, over 12,000 man-made fluorine-containing chemicals are categorized as PFAS [1]. The chemical and thermal stability of PFAS, attributed to the robust carbon–fluorine bond, renders them resistant to environmental degradation [2]. Additionally, their hydrophobic and hydrophilic properties make them widely used as surfactants in consumer goods [3]. Polyfluoroalkyl species, characterized by at least one perfluoroalkyl group, have at least one carbon atom that cannot bond with a fluorine atom [4]. Under certain conditions, these species can transform into perfluoroalkyl species through either nonbiological or biological pathways, as evidenced or theorized [2]. Long-chain PFAS refer to perfluoroalkyl sulfonates (PFSAs) with at least six carbon atoms and perfluoroalkyl carboxylic acids (PFCAs) with at least eight carbon atoms [5]. Owing to their higher bioaccumulative potential, long-chain PFAS represent significant health risks to humans and other organisms [6]. Legacy per- and polyfluoroalkyl substances (PFAS) encompass a range of compounds, notably perfluorobutane sulfonate (PFBS), perfluorooctanoic acid (PFOA), perfluorooctane sulfonate (PFOS), perfluorononanoic acid (PFNA), perfluorohexane sulfonate (PFHxS), perfluorodecanoic acid (PFDA), perfluoroheptanoic acid (PFHpS), perfluoroundecanoic acid (PFUnA), and perfluorododecanoic acid (PFDoA) [7]. Owing to their persistence and biological toxicity, the Stockholm Convention on Persistent Organic Pollutants has been amended to incorporate traditional PFAS, specifically PFOA, PFOS, and PFHxS. Consequently, these substances are now subject to restrictions or discontinuation [8]. Emerging alternatives to PFOS include polyfluoroalkyl ether sulfonic acids (PFESA) and Sodium p-perfluorous nonenoxybenzene acids sulfonate (OBS), while polyfluoroalkyl ether carboxylic acid (PFECA), perfluoro-2-propoxypropionic acid (HFPO-DA), 6:2 fluorotelomer carboxylic acid (6:2 FTCA), and 4,8-dioxa-3H-perfluorononononononylammonium acid (ADONA) have been identified as alternatives to PFOA [9,10]. The potential for toxicity of these novel PFAS compounds necessitates further research. It is urgent to improve the awareness of the potential threat of PFAS to human health.

Acting as endocrine-disrupting chemicals (EDCs), PFAS have been shown to negatively affect reproductive health, leading to potential female infertility and adverse pregnancy outcomes (Table 1). Initially, exposure to PFAS can induce disturbances in reproductive endocrine and metabolic functions. PFAS can inhibit the activity of the reproductive axis, leading to metabolic disorders involving gonadotropins and sex hormones [11,12,13,14,15,16,17].

**Figure 1:**
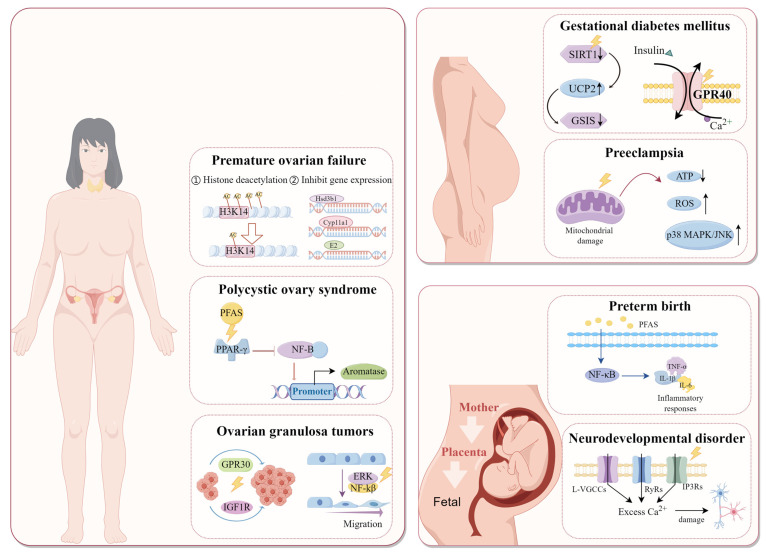
**Potential mechanisms by which PFAS impair female reproductive health.** Premature ovarian insufficiency-related mechanisms can be found in Section 5.1.1; polycystic ovary syndrome-related mechanisms can be found in Section 5.1.2; ovarian tumor-related mechanisms can be found in Section 5.2.2; gestational diabetes-related mechanisms can be found in Section 7.1; preeclampsia--related mechanisms can be found in Section 7.2; preterm birth-related mechanisms can be found in Section 8.1; neurodevelopmental abnormalities-related mechanisms can be found in Section 8.2. PPAR: peroxidase-activated proliferative receptor, NF-B: nuclear factor-B, GPR30: G-protein-coupled receptor 30, IGF1R: insulin-like growth factor 1 receptor, ERK: extracellular regulated protein kinases, SIRT1: silent information regulator 1, UCP2: uncoupling protein 2, GSIS: glucose-stimulated insulin secretion, GPR40: G-protein-coupled receptor 40, ROS: reactive oxygen species, MAPK: mitogen-activated protein kinase, JNK: c-Jun N-terminal kinase, TNF-α: tumor necrosis factor-α, IL-1β: interleukin-1β, IL-6: interleukin-6, L-VGCCs: L-type voltage-gated calcium channels, RyRs: ryanodine receptors, IP3Rs: inositol 1,4,5-triphosphate receptor-mediated (created using figdraw).

Given the growing significance of the correlation between environmental pollutant exposure and female reproductive health, providing an overview of PFAS’s impact on female reproductive health and their mechanisms is essential for comprehending trends in female reproduction, associated diseases, and for establishing pertinent environmental measures. However, a comprehensive analysis detailing how PFAS affect women’s reproductive health across different phases is still lacking. This review specifically summarizes the sources and pathways of PFAS, with a focus on pre-pregnancy, mid-pregnancy, and pregnancy outcomes. Special attention is given to the impact of PFAS on the reproductive health of women of childbearing age, identifying research gaps and contradictory findings. Moreover, understanding the molecular targets of PFAS in female reproductive tissues is emphasized to elucidate the mechanisms of PFAS-related reproductive toxicity and to aid in developing therapies to enhance women’s reproductive health (Figure 1).

## 2. Review Scope and Methodology

In this review, we searched for publications in Google Scholar and PubMed using the following search terms: “Per- and Polyfluoroalkyl Substances” or “PFAS” along with “female reproductive health” or “reproductive toxicity” or “infertility” or “adverse pregnancy outcomes” or “PCOS” or “premature ovarian failure”. Studies that met the following criteria were included in Table 1: (1) cohort or case–control studies with full texts available; (2) studies measuring exposure to PFAS in biological samples, such as plasma, serum, or amniotic fluid from women; (3) studies that provided quantitative estimates of the relationship between exposure to PFAS and reproductive health, including odds ratios (OR) from Cox models or logistic regression models and their 95% confidence intervals (CI). Systematic reviews, editorial materials, letters, and conference abstracts were excluded.

## 3. Sources and Methods of Human Exposure to PFAS

Extensive evidence indicates that individuals can ingest PFAS through various methods [35,36]. Occupational exposure primarily occurs through inhalation and skin contact [37]. In the general population, PFAS exposure routes include drinking water, food, and indoor air [38,39,40]. Household items such as decor, carpets, paper goods, building materials, impregnating agents, detergents, and cosmetics also contain PFAS [39]. Some PFAS polymers can volatilize, allowing bodily penetration via skin contact or inhalation [41,42]. Drinking water consumption is considered the primary route of PFAS exposure, as these substances have been found to be detectable in both surface and drinking water near contaminated sites, particularly those associated with fluoropolymer manufacturing plants [43,44]. Plants, sheep, and cattle that consume surface water contaminated with PFAS can accumulate these compounds in their bodies. This can result in the contamination of agricultural products such as meat and dairy products [45]. Among food sources, seafood is the primary source of PFAS exposure in humans, while dairy products, meat, and eggs are also routes of exposure that may be linked to bioaccumulation in the food chain [39,46]. PFAS can also migrate from food contact materials (FCMs) into food, posing dietary risks. Such materials include microwave popcorn bags, fast food packaging, and non-stick cookware [47,48].

## 4. Metabolic Pathways of PFAS in Humans

The transport, accumulation, and metabolic clearance of PFAS in humans depend on high protein affinity [49]. Due to their extremely hydrophobic nature, PFAS have a strong affinity for binding to various transporters in the body. This includes serum proteins, fatty acid transporters, and organic anion transporters [50]. Target proteins’ binding cavities are occupied by the hydrophobic fluorinated carbon chains of PFAS, and their acid moieties establish hydrogen bonds with amino acid residues there [50]. Additionally, PFAS can passively penetrate phospholipid bilayers, shrinking lipids while also making biofilms more fluid and disrupting their structure [51]. The primary methods through which PFAS are excreted in humans is by urine clearance [52]. According to a study, the geometric mean urinary elimination rates of PFOS and PFOA in adults were 16% and 25%, respectively [53]. Biliary clearance and fecal excretion serve as additional PFAS elimination routes [54,55]. For women, menstruation, pregnancy, and lactation are specific routes of elimination of PFAS, explaining the difference in elimination of PFAS between men and women [56,57]. The loss of blood during menstruation facilitates PFAS excretion, given the similar albumin content between menstrual and whole blood [58]. Factors such as heavy menstrual bleeding, use of oral contraceptives, irregular menstrual cycles, and extended periods can affect PFAS elimination in the preconception phase of females [59]. Additionally, lactation and placental transfer are critical for maternal PFAS excretion and neonatal exposure [60,61,62,63].

## 5. The Effect of PFAS Exposure on the Reproductive Health of Females of Childbearing Age

### 5.1. Reproductive Endocrine Disruption

#### 5.1.1. Ovarian Dysfunction

Female ovarian dysfunction is a progressive condition, characterized by three stages: diminished ovarian reserve (DOR), premature ovarian insufficiency (POI), and premature ovarian failure (POF). DOR is marked by a reduced capacity of the ovaries to produce eggs and a decline in follicle quality. POI involves follicle depletion and ovarian hypofunction before the age of 40, while POF, the final stage of POI, is identified by amenorrhea, decreased estrogen levels, and follicle-stimulating hormone (FSH) levels exceeding 40 IU/L in women under 40, accompanied by varying degrees of perimenopausal symptoms [64]. The ovarian reserve dwindles as most follicles undergo activation and atresia, leaving only a few follicles as menopause approaches. The onset of POI or POF can be attributed to environmental, genetic, autoimmune, developmental factors, or medical/surgical interventions [65]. Given that infertility resulting from these conditions remains a challenge for assisted reproductive technologies, it is imperative to explore the role and mechanisms of various risk factors in ovarian dysfunction to avert its development.

A single-center prospective study identified PFOA as a potential factor contributing to DOR. This was supported by non-targeted metabolomic analysis of follicular fluid from DOR patients, revealing significant alterations in 12 metabolites in the high concentration group compared to the low concentration group [66]. In a Chinese case–control study, Zhang et al. [12] evaluated the association between serum PFAS levels and POI. The findings indicated that elevated concentrations of PFOS, PFOA, and PFHxS were associated with an increased risk of POI, while no significant associations were found for six other PFAS compounds (PFNA, PFBS, PFUA, PFHpA, PFDeA, and PFDoA). In addition, animal studies found that in neonatal female rats exposed to PFOS or PFOA, despite the fact that the ovarian shape did not change, the corpus luteum and ovarian primordial follicle count were considerably decreased [67].

The underlying mechanism might involve a reduction in histone H3K14 acetylation at the steroidogenic acute regulatory protein (StaR) promoter site in female mice with chronic exposure to PFOS, leading to inhibited estrogen synthesis, impaired follicle development and ovulation, and a reduced ovarian follicular reserve [68]. These results suggested that PFOA may affect the ovarian reserve function by changing the metabolic composition of follicular fluid [66]. Several researchers conducted in vitro and in vivo studies in mice to investigate the effects of PFOA on hormone levels, folliculogenesis, and ovarian steroid gene expression. In vitro, PFOA significantly decreased the gene expression of Cyp11a1, Hsd3b1 and StaR, estradiol (E2), and estrone concentration, and inhibited follicle growth compared with the control group. In vivo, compared with controls, exposure to PFOA (5 mg/kg) increased gene expression of Cyp19a1 and the antral follicle count, and decreased primitive follicle counts, suggesting that PFOA may accelerate follicle formation [69]. Accelerated folliculogenesis may result in POI due to primordial follicle pool renewal failure. At the same time, PFOA can cause oocyte degeneration by influencing mitochondrial malfunction and death in children, according to a single-cell transcriptome study [70]. In conclusion, PFOA may impair ovarian function and increase the risk of ovarian dysfunction through a non-monotonic mechanism.

#### 5.1.2. Polycystic Ovary Syndrome (PCOS)

PCOS is a common disorder affecting females of reproductive age, characterized by symptoms including oligomenorrhea, infertility, hirsutism, acne, and obesity [71]. Moreover, PCOS is associated with an increased risk of pregnancy-related complications, such as type 2 diabetes [72], adverse pregnancy outcomes, and gestational hypertension [73]. Globally, PCOS affects approximately 15% of women, imposing a significant economic burden [74].

Recent epidemiological studies have demonstrated a correlation between PCOS and exposure to PFAS. Notably, a study from the United States provided initial evidence that higher serum levels of PFOS and PFOA are associated with an increased risk of PCOS [75]. In a different study, individuals with PCOS had greater serum PFOS concentrations than controls, and in the control group, higher serum PFOS concentrations were linked to irregular menstruation [13]. Despite PFOS and PFOA being linked to an increased risk of PCOS, no correlation has been found between these PFOA, PFOS, and PCOS-related infertility. A study, controlling for factors such as body mass index and age, discovered a dose-dependent correlation between PFDoA, PFUnDA exposure, and the incidence of PCOS-related infertility [32].

Although the exact pathogenesis of PCOS remains unclear, there is growing proof that the development of PCOS is driven by changes in the epigenetic and developmental program caused by imbalances in the maternal uterine environment [76]. Following the stimulation of luteal and granulosa cells from porcine ovarian tissue with 500 ng/mL of FSH or luteinizing hormone (LH), these cells were isolated in vitro and exposed to 1.2 μM of PFOS or PFOA. It was found that perfluorinated substances inhibited the secretion of progesterone, E2, and androstenedione in both cell types [77]. In addition, PFAS, as an endocrine disruptor, can activate peroxidase-activated proliferative receptors (PPARs) [78], and PPAR-γ may interfere with the interaction between nuclear factor-B (NF-B) and aromatase promoter II (ArpII) to prevent the expression of aromatase, which controls the transformation of androgens into estrogens [79]. This could be one of the potential mechanisms by which PFAS promote the development of PCOS.

### 5.2. Effect of PFAS Exposure on Female Reproductive Tract

#### 5.2.1. Endometriosis

Endometriosis, characterized by the proliferation of endometrial glands and stroma beyond the uterine confines, represents a prevalent estrogen-dependent gynecological disorder. The primary clinical symptoms include dysmenorrhea and chronic pelvic pain [80]. It is a significant contributor to infertility, implicated in 30–50% of cases among females [81]. While the precise etiology of endometriosis remains elusive, recent investigations have pivoted towards the role of environmental endocrine disruptors. PFAS, known to modulate gene expression linked to endocrine function and estrogen receptors, display estrogen-like effects in vitro, potentially influencing the reproductive system in animals [82]. This suggests a biologically plausible link between PFAS exposure and the development of endometriosis, a condition intrinsically tied to hormonal regulation.

In 2012, research first established a link between two PFAS—PFNA and PFOA—and elevated odds of endometriosis diagnosis [29]. In this research, mean concentrations of PFNA and PFOA in groups of women with endometriosis were 0.69 and 2.65 ng/mL, which were lower than the levels found in American women based on biomonitoring data. Similarly, another US study identified a positive association between PFOS, PFOA, and PFNA levels and endometriosis. [24]. This study reported the following mean PFAS concentrations in women with endometriosis: 16.28 ng/mL for PFOS, 3.48 ng/mL for PFOA, and 1.00 ng/mL for PFNA. A case–control study among Chinese women investigated the association between PFAS exposure and infertility related to endometriosis. The study found that higher plasma levels of PFBS were associated with increased odds of infertility due to endometriosis [18]. However, some studies have yielded inconsistent results. Matta et al.’s study [83] identified a connection between persistent organic pollutants and endometriosis through metabolic and cytokine profile analysis. Nevertheless, it did not find evidence linking PFAS (including PFHpS, PFOS, PFOA, PFNA, PFDA, and PFUnA) to an increased risk of endometriosis. In this study, median concentrations of PFASs in groups of women with deep endometriosis without endometrioma were reported as follows: 0.05 ng/mL for PFHpS, 2.09 ng/mL for PFOS, 1.21 ng/mL for PFOA, 0.48 ng/mL for PFNA, 0.22 ng/mL for PFDA, and 0.15 ng/mL for PFUnA. Variations in PFAS concentrations among the study populations may account for the contradictory findings. A meta-analysis is warranted to more definitively ascertain the association between PFAS exposure and endometriosis.

Despite the growing body of evidence linking PFAS exposure to endometriosis, the underlying biological mechanisms have yet to be fully elucidated. It can be speculated that the estrogenic properties of PFAS may be one of the mechanisms. In addition, PFAS has cytokine inhibitory and inflammatory effects [84], which may also be an underlying mechanism of endometriosis [80]. The precise molecular targets of PFAS eagerly need to be investigated in future studies by utilizing large-scale profiling techniques such as proteomics, transcriptomics, or metabolomics.

#### 5.2.2. Reproductive Tract Tumors

PFAS, as estrogen analogs, can bind to estrogen receptors, partially blocking them and activating their response [85]. This interference restricts genome regulation and increases the risk of developing ovarian cancer. Studies have shown that there are variations in dose–response relationships for different types of PFAS. For instance, each unit increase in PFOA concentration leads to a 2% increase in the likelihood of ovarian cancer; while each unit increase in PFOS concentration results in a 1% increase in the likelihood of ovarian cancer. Additionally, each unit increase in PFHS raises the risk of ovarian cancer by 3%. Furthermore, for each unit increase in PFDE, the incidence of ovarian cancer increases by 29% [25]. The potential mechanism may involve GPR30 and Insulin-like growth factor 1 receptor (IGF1R) as mitogenic factors in granulosa cells responding to the combination of PFOA and PFOS in human follicular fluid, promoting the growth of ovarian granulosa tumors [86,87]. Moreover, PFOA can stimulate the migration and invasion of ovarian cancer cells by activating the regulated protein kinases (ERK)/nuclear transcription factor-κB (NF-κB) signaling pathway [88].

The association between PFAS exposure and the risk of uterine cancer has also attracted attention. Research indicates that different types of PFAS exhibit varying dose–response relationships with uterine cancer. For example, each unit increase in PFOS concentration decreases the risk of uterine cancer by 5.5%. Conversely, for PFNA, each unit increase reduces the incidence of uterine cancer by 57.5%. Additionally, each unit increase in PFHS decreases the risk of uterine cancer by 39.2% [25]. These findings underscore the potential impact of PFAS on the development of uterine cancer, necessitating further research to understand the specific mechanisms involved.

## 6. The Association between PFAS and In Vitro Fertilization (IVF) Outcomes

Previously, numerous studies have been cited that investigate the impact of PFAS on the reproductive health of females of reproductive age, focusing on conditions such as POI, PCOS, thyroid dysfunction, and endometriosis, all of which contribute to increased infertility rates. Consequently, individuals attempt to address disease-related infertility associated with PFAS exposure through assisted reproductive technologies. However, Kim et al. [89] identified the presence of PFAS in the follicular fluid of patients undergoing IVF treatment. For the production of fully competent oocytes, a balanced interplay among hormones, growth factors, oocytes, and surrounding somatic cells is essential. However, PFAS, acting as endocrine disruptors, may directly compromise the fertilization capability of oocytes [90], potentially resulting in IVF failure. A significant cohort study in China investigated the link between IVF outcomes and PFAS concentrations in follicular fluid, finding that exposure to nine different PFAS mixtures was associated with a decreased frequency of high-quality embryos, potentially diminishing IVF success rates [91]. McCoy et al. [92] observed no correlation between PFAS exposure levels and overall fertility rates or pregnancy outcomes. However, they noted a negative correlation between the blastocyst conversion rate and the levels of PFDA and PFUnA in follicular fluid. Similarly, Ma et al. [93] discovered an inverse relationship between the maternal plasma concentration of PFOA and the number of retrieved oocytes, mature oocytes, 2 PN zygotes, and high-quality embryos. Nonetheless, no PFAS were significantly associated with the chances of implantation, clinical pregnancy, or successful delivery.

Interestingly, the existing studies have yielded inconsistent conclusions regarding the impact of PFAS on IVF outcomes. A cohort study from Belgium revealed that elevated PFAS levels in follicular fluid correlated with higher conception rates and an increased number of high-quality embryos [94], challenging the prevailing assumption that PFAS decreases female fertility. The divergent findings on the correlation between PFAS exposure levels and IVF outcomes may stem from several factors, including the small sample sizes of most studies and differences in demographic characteristics among study populations.

## 7. Effect of PFAS Exposure on Maternal Health during Pregnancy

### 7.1. Gestational Diabetes Mellitus (GDM)

GDM, characterized by transient glucose intolerance during pregnancy, is a common complication that increases in prevalence with maternal age [95]. GDM can lead to adverse birth outcomes [96] and increases the odds of fetal birth defects [97]. Factors including pre-pregnancy obesity, high carbohydrate diet, advanced maternal age, and a family history of Type 2 diabetes are linked to an elevated risk of GDM [98], though the precise mechanism is yet to be elucidated.

Despite the growing body of research on the relationship between PFAS and GDM, findings remain contradictory. Epidemiological evidence suggests that exposure to PFOA and PFOS during pregnancy may disrupt glucose homeostasis through impairment of maternal thyroid or liver function, thereby increasing GDM risk [95]. Cohort studies have consistently demonstrated a positive association between exposure to PFOA, PFOS, or PFAS mixtures and GDM [99,100,101]. Similarly, a meta-analysis of eight studies found a significant association between PFOA exposure and GDM, whereas exposure to other PFAS types, including PFHxS, PFOS, and PFNA, did not show a statistically significant correlation with GDM risk [84]. The effects of various PFAS types on health outcomes may exhibit directional heterogeneity. Wang et al. found that PFOA concentrations were significantly associated with insulin resistance (IR), oral glucose tolerance test (OGTT) results, and GDM, whereas serum PFOS levels were inversely associated with GDM [102]. A case–control study in Shanghai, China, reported that elevated first-trimester maternal blood levels of PFBS and PFDoA were significantly associated with increased GDM risk, whereas PFOS and PFOA levels were not statistically significant predictors of GDM [19]. Concurrently, one study indicated that while certain exposures to PFAS were significantly associated with elevated blood sugar levels, they did not correlate with increased GDM risk [103]. In summary, exposure to PFAS may differentially affect glucose homeostasis and GDM risk in pregnant women, potentially due to variations in PFAS type, exposure levels, and interactions. Further confirmation of the PFAS-GDM association requires meta-analytical review.

The underlying mechanism of PFAS causing GDM remains unclear, but PPARs are the metabolic pathways that have attracted the most interest. PFAS can stimulate PPAR-α expression [104], which can decrease hepatic glucose uptake, inhibit the conversion of pyruvate to acetyl-CoA, and enhance gluconeogenesis, thus disrupting glucose homeostasis [105]. Additionally, PFAS can disrupt thyroid hormone (TH) homeostasis during pregnancy through increased thyroid-stimulating hormone (TSH) and decreased T3 and T4 levels, thereby impairing glucose metabolism and elevating GDM risk [85]. While prolonged PFOS exposure did not significantly change insulin gene expression, it inhibited silent information regulator 1 (SIRT1) activity and increased uncoupling protein 2 (UCP2) expression [106]. This suggests that PFOS-induced impairment in glucose-stimulated insulin secretion (GSIS) is mediated, at least in part, by the SIRT1-UCP2 pathway [106]. Furthermore, Qin et al. demonstrated, using a mouse model, that one-hour exposure to PFOS activates G-protein-coupled receptor 40 (GPR40), leading to increased intracellular calcium levels and insulin release. This offers evidence of a novel mechanism through which PFAS disrupt insulin secretion [107].

### 7.2. Hypertensive Disorders of Pregnancy (HDP)

HDP are defined as hypertension that arises during pregnancy. The four primary forms of HDP include preeclampsia–eclampsia, chronic hypertension, gestational hypertension, and superimposed preeclampsia on chronic hypertension [108]. Multiple cohort studies have demonstrated a correlation between higher PFAS exposure and increased risk of preeclampsia [109,110,111,112]. However, the Bayesian Kernel Machine Regression (BKMR) analysis in the Viva project found significant correlations between plasma concentrations in PFAS mixtures and pregnancy-related hypertension, but not preeclampsia [113]. A prospective pregnancy cohort study, after adjusting for potential confounders, linked elevated PFOS and PFNA levels at 10 weeks of gestation to preeclampsia, whereas PFOA did not show a statistically significant correlation [26]. The Canadian MIREC study found that high levels of PFHxS were associated with preeclampsia compared to gestational hypertension, whereas PFOS and PFOA showed no association with either condition, and fetal sex may influence this relationship [21]. Different subtypes of preeclampsia have distinct mechanistic bases: early-onset preeclampsia is attributed to defects in placental formation, whereas late-onset preeclampsia is associated with placental aging, maternal genetic predispositions to cardiovascular and metabolic diseases, and their interactions [114]. Consequently, several studies have sought to elucidate the relationship between PFAS exposure and preeclampsia by examining disease subtypes. A study found that first-trimester plasma levels of PFDA and PFOS were associated with an increased risk of late-onset preeclampsia, but not with early-onset preeclampsia [14]. Conversely, Tian et al. [109] reported a significant correlation between early-onset preeclampsia and concentrations of PFUnDA and PFOA, with no significant effect on late-onset preeclampsia.

Preeclampsia is partially attributed to inadequate trophoblast invasion of the decidua [115]. Consequently, environmental pollutants that disrupt invasion pathways may contribute to the development of preeclampsia. The adverse effects of PFOS exposure on trophoblast cell migration and invasion may also stem from mitochondrial damage, evidenced by reduced ATP production, increased reactive oxygen species (ROS) generation, and diminished mitochondrial membrane potential, alongside the activation of the p38 Mitogen-activated protein kinase (MAPK) and c-Jun N-terminal kinase (JNK) signaling pathways [116]. Furthermore, PFOA exposure resulted in an increased abundance of the NOTCH intracellular domain (NICD) in HTR8/SVneo cells [117]. Given that Notch signaling plays a role in trophoblast migration, this mechanism may be implicated in the development of preeclampsia [118].

### 7.3. Abnormal Thyroid Function

In addition to the hypothalamic–pituitary–ovarian axis, the thyroid axis is integral to the normal maintenance of female reproductive function. Due to its direct and indirect interactions with the hypothalamic–pituitary–ovarian axis and reproductive systems, severe thyroid disease can lead to irregular menstruation and infertility [119]. Consequently, comprehending PFAS-induced alterations in thyroid function is vital for female reproductive health.

An increasing number of epidemiological studies are currently focusing on the correlation between PFAS and thyroid dysfunction in pregnant women. Project Viva in the US observed a negative correlation between concentrations of PFHxS, PFOA, and MeFOSAA and free thyroxine 4 (FT4) levels in maternal plasma collected cross-sectionally during the first trimester; however, it found no statistically significant correlation between PFAS concentrations and maternal T4 levels [120]. In a cross-sectional study from Spain, a significant positive association was found between PFHxS and TSH levels in pregnant women, as well as a negative correlation between PFNA, PFOA, and TT3 concentrations, along with iodothyronine deiodinase 1-CC (DIO1-CC) [121]. In contrast, a Shanghai birth cohort study examining blood PFAS and thyroid hormone concentrations in pregnant women before 16 weeks found positive associations between PFOA, PFHxS, PFNA, and levels of FT4 or FT3. Conversely, PFHxS showed a negative correlation with TSH, and the presence of thyroid peroxidase antibodies (TPOAb) appeared to alter the correlation between these PFAS and thyroid hormones (THs). In this study, the median concentrations of PFASs in maternal serum were reported as follows: 0.54 ng/mL for PFHxS, 1.63 ng/mL for PFNA, 12.32 ng/mL for PFOA, 0.16 ng/mL for PFDoA, and 9.25 ng/mL for PFOS [122]. More recently, A Canadian cohort study revealed no association between PFASs (PFHxS, PFNA, PFOA, and PFOS) and FT4, total thyroxine (TT4), or TSH levels in women with normal TPOAb. However, in the subset of women with elevated TPOAb levels (9% of the cohort, n = 14), increases in PFNA, PFOA, and PFOS within the interquartile range were linked to a significant elevation in maternal TSH levels, ranging between 46% and 69%, with 95% confidence intervals (CIs) extending from 8% to 123%. In the study, the median concentrations of PFASs in maternal serum were reported as follows: 1.0 ng/mL for PFHxS, 0.6 ng/mL for PFNA, 1.7 ng/mL for PFOA, and 4.8 ng/mL for PFOS [123]. This suggests that TPOAb may be an effector mediator of the association between PFAS and THs and that the thyroid-damaging effects and susceptibility to PFAS exposure vary according to maternal TA levels [124]. Although the above studies reported positive links between maternal PFOA levels and T4 levels, other studies found that Low T4 levels were connected to high PFNA, PFUdA, and PFDOA concentrations [125]. Different PFAS have diverse effects on FT4, indicating that their impact on thyroid function varies with the specific PFAS, exposure duration, and concentration.

Multiple mechanisms could explain the observed association between PFAS levels and serum thyroid-associated hormones. Toxicological studies indicate that PFOS-induced hypothyroxinemia is partly due to increased hepatic T4 glucuronidation via uridine diphosphoglucuronosyl transferases (UGT1A1) and thyroidal T4 to T3 conversion by type 1 deiodinase (DIO1) [126]. Additionally, PFAS can bind to transthyretin (TTR) and thyroid hormone receptors (TRs), potentially leading to increased excretion of T4 and T3 and disrupting TH homeostasis [127,128]. Disruption of iodine homeostasis in thyroid cells may also play a role in PFOS’s impact on thyroid function. Conti [129] utilized live-cell imaging to monitor intracellular iodine content fluctuations with the genetically encoded halide-sensitive biosensor YFP-H148Q/I152L. This research indicates that PFOS can inhibit the NIS-mediated uptake of iodine, leading to reduced intracellular iodine levels in iodine-containing cells. This inhibition results in impaired iodine accumulation in FRTL-5 thyroid cells, disrupting iodine homeostasis.

### 7.4. Other Pregnancy Complications

In addition to the adverse effects mentioned above, PFAS may also cause anemia and gestational weight gain (GWG) in pregnant females. A recent study indicated that maternal exposure to PFDoA elevated the risk of gestational anemia in early pregnancy, whereas PFBS exposure reduced this risk in the second trimester. Furthermore, a mixture of PFAS was not significantly associated with gestational anemia [33]. Cui et al. [99] posited that iron supplementation might amplify the effects of PFAS exposure on hematocrit (HCT) and hemoglobin (Hb) levels. The presence of PFAS may reduce Hb’s oxygen-binding capacity, potentially leading to an increase in Hb levels.

In the Project Viva cohort study, exposure to perfluoroalkyl substances (PFAS) was linked to GWG [130]. Specifically, PFOA exhibited a positive association with GWG in overweight women, while PFOS and PFHxS were inversely associated with GWG [131].

## 8. Adverse Pregnancy Outcome

### 8.1. Preterm Birth

Preterm birth is defined by the WHO as any delivery that occurs before 37 weeks of pregnancy or less than 259 days following the start of a woman’s last menstrual cycle. Preterm delivery complications are particularly detrimental to newborns and children under the age of five [132]. Preterm birth has been linked to an increased risk of exposure to EDCs [133,134]. However, the epidemiological evidence linking PFAS exposure to preterm birth is inconsistent. A cohort study in coastal China indicated that elevated concentrations of PFAS mixtures were associated with increased risks of preterm birth at household levels. Specifically, increased exposures to PFDA and PFNA were associated with higher odds of preterm delivery [135]. Another study found that prenatal exposure to a combination of PFAS increased the odds of preterm delivery, with risks positively correlated with PFOS, PFHpA, PFBS, and PFSA levels. Conversely, the presence of PFHxS, PFOA, and PFUnA was inversely associated with the risk of preterm delivery [20]. This aligns with the findings from a delivery cohort study, which reported approximately a 2-fold increase in the odds of preterm delivery in quartiles with higher PFOS and PFOA concentrations [27]. According to a cohort study in Taiwan, PFOS levels in cord plasma were inversely related to the risk of preterm birth [136]. Comprehensive analysis revealed a non-linear relationship between PFNA and PFOA levels and preterm delivery, and a linear positive relationship between PFOS levels and the odds of preterm delivery. The non-linear associations between PFNA and PFOA levels and preterm birth may be attributable to threshold effects [16]. Contrary to previous studies, Huo’s research indicated that only increases in PFNA quartiles were associated with preterm delivery, while PFAS exposure generally showed no relation to gestational age at birth or birth weight. Several logistic models revealed no association between PFAS exposure and total preterm birth, spontaneous preterm birth, or suggestive preterm birth [137]. A cohort study in Spain found no significant association between PFOS and PFOA levels during the first trimester of pregnancy and birth outcomes [138]. In the C8 Health Project, Darrow et al. [139] similarly concluded that no significant correlation existed between serum PFOA or PFOS levels and the incidence of preterm birth. These findings suggest that further epidemiological evidence is still needed for the relationship between PFAS exposure and the odds of preterm delivery.

Inflammation serves as a physiological mechanism at the onset of term pregnancy, and the inability to establish immune tolerance or suppress excessive inflammation can result in preterm birth [140]. Intrauterine inflammation can trigger delivery regardless of gestational age and fetal maturity, even without the presence of infection [141]. Previous reports have indicated that PFAS can induce inflammatory responses. When macrophages phagocytose PFOS, it activates the NF-κB signaling pathway, leading to the secretion of several pro-inflammatory cytokines such as tumor necrosis factor-alpha (TNF-α), interleukin-1 beta (IL-1β), and interleukin-6 (IL-6) [142]. This hyperinflammatory state could contribute to the mechanism behind PFAS-induced preterm birth. Although continuous exposure to low levels of PFAS may not directly cause preterm birth, it is associated with the induction of inflammation [143].

### 8.2. Pregnancy Loss

Miscarriage, also referred to as abortion, is defined as the loss of a pregnancy before 20 or 22 weeks of gestation. Miscarriage represents a frequent complication during early pregnancy stages [144]. Recurrent spontaneous abortions (RSA) are characterized by three or more miscarriages occurring between 20 and 28 weeks of pregnancy [145]. Common risk factors for miscarriage include chromosomal abnormalities, maternal age and BMI, lifestyle factors, and endometrial conditions [144]. Many EDCs have been shown to be associated with the development of miscarriage [146]. EDCs may cause X chromosome aneuploidy in oocytes and increase the risk of chromosome loss and infertility, autoimmune diseases, cancer, and various genetic disorders [147]. A Swedish cohort study demonstrated a significant association between early-pregnancy blood levels of PFOA and increased likelihood of spontaneous miscarriage in the second trimester. Higher maternal plasma concentrations of PFHpS and PFOA were discovered in a case–control study of DNBC (samples from 1996 to 2002), which is most consistent with an increased danger of miscarriage in multiparous women between weeks 12 and 22 of pregnancy. Increasing concentrations of PFOA and PFOS were associated with a monotonic increase in the risk of miscarriage. The odds ratios (ORs) for the highest versus lowest quartiles of PFOA or PFHpS exposure were 2.2 (95% CI: 1.2–3.9) and 1.8 (95% CI: 1.0–3.2), respectively. This PFAS exposure has been more consistently associated with an elevated risk of miscarriage in multiparous women, possibly due to placental transfer serving as a pathway for maternal PFAS excretion [23]. However, another study from Denmark (enrolling pregnant women from 2010 to 2012) did not explore the relationship between PFOA and miscarriage, but serum PFHxS, PFDA, and PFNA were positively related to miscarriage. After adjusting for parity, BMI, age, and gestational age at the time of serum collection, the adjusted ORs for miscarriage were 16.17 (95% CI: 6.88–38.03) for the highest tertile of PFDA exposure and 2.67 (95% CI: 1.31–5.44) for PFNA, respectively. One explanation would be that PFOA was linked to a rise in PFHxS but a drop in PFOS in Denmark after 2010 [31]. Darrow et al. identified a strong association between miscarriage and PFOS exposure in nulliparous women (aOR = 1.34, 95% CI: 1.02–1.76) (aOR = 1.34, 95% CI: 1.02–1.76) [28].

The pathogenesis of RSA remains very complex. While the pathogenesis of RSA primarily centers on immunity and genetics, the impact of environmental exposure remains unclear. The emerging PFAS substitutes, HFPO-DA and 6:2 Cl-PFESA, have been associated with risks of unexplained RSA, particularly in older women. The likely mechanism is that 6:2 Cl-PFESA has a median elimination half-life of up to 15.3 years and may be more bioaccumulative than PFOS. HFPO-DA, on the other hand, may lead to cell damage by stimulating ROS generation [34]. Nonetheless, research into the reproductive toxicity of PFAS on RSA is limited, necessitating further investigation into the reproductive toxicity of emerging PFAS substitutes.

### 8.3. Low Birth Weight

Infant weight is a critical metric for assessing health, impacting both growth and mental development. The World Health Organization defines low birth weight (LBW) as a birth weight of less than 2500 g, a significant predictor of infant mortality and morbidity [148,149].

Evidence suggests that PFAS influence fetal growth and development. An analysis of 98 umbilical cord samples from Hangzhou, China, revealed a negative correlation between PFOS exposure and birth weight [150]. One study found that increased prenatal PFAS exposure was inversely associated with birth weight z-scores, particularly in individuals with elevated maternal glucose levels. Elevated blood glucose, which can alter the structure and function of the placenta, may increase its permeability to PFAS due to their ability to translocate across the placenta [151]. A retrospective study including 105,114 singleton live births in Veneto Region, Italy, showed a significant association between PFAS exposure in contaminated areas and fetal growth and development. Living in PFAS-contaminated areas was associated with a higher probability of having small-for-gestational-age infants (adjusted OR = 1.27, 95% CI: 1.16–1.39) [152]. A comprehensive review and meta-analysis revealed no connection was found between pregnant PFOA exposure and low newborn weights in kids (OR = 0.90, 95% CI: 0.80–1.01). The connection between PFOS exposure during pregnancy and low birth weight in infants, however, was significantly favorable (OR = 1.32, 95% CI:1.09–1.55) [153]. A Shanghai birth cohort revealed that early pregnancy exposure to PFOS, PFDA, PFNA, PFUA, and PFDoA was strongly linked to shorter birth length, especially in mothers of normal weight and female fetuses, but not to birth weight [154].

The maternal thyroid is essential for controlling fetal growth and development throughout pregnancy. The maternal thyroid hormone facilitates fetal growth by promoting placentation, regulating metabolism, stimulating fetal oxygen consumption, and influencing the hormones and growth factors directly involved in fetal development [155,156]. During the early stages of pregnancy, the fetus is almost entirely dependent on the maternal thyroid hormone. The fetal hypothalamic–pituitary–thyroid (HPT) system starts functioning during the second trimester and matures at 35 weeks of gestation [157]. PFAS are detrimental to the thyroid gland because they can inhibit the thyroid hormone from binding to its receptors [158]. Pre-pregnancy maternal exposure to PFAS disrupts maternal thyroid hormone production, leading to inadequate placental transfer of thyroid hormone and adversely affecting fetal growth and development. PFAS can also have toxic effects on the fetal thyroid gland through direct placental transfer (refer to Section 8.4 for a detailed description). Consequently, thyroid hormones may be a contributing factor to LBW. Estrogen also plays a role in affecting fetal growth and development [159,160]. PFAS exposure affects estrogen receptor activity, expression, and estrogen levels, thereby impacting fetal development [161]. Additionally, PFAS activation of peroxisome proliferator-activated receptors (PPARs) has been observed in both human and animal models. PFAS may disrupt placental function through PPAR signaling, causing dysregulation of placental lipid homeostasis and resulting in fetal developmental restrictions [60].

### 8.4. Fetal/Neonatal Thyroid Dysfunction

Normal embryonic and fetal development is dependent on thyroid hormones. In the early trimester of pregnancy, the fetus is almost entirely reliant on maternal THs. THs are crucial for the cognitive and neurodevelopment of the fetus, particularly in early pregnancy [162]. Thyroid function in fetuses does not develop until 11–12 weeks of pregnancy [163]. T4 can be activated by deiodinase to T3 to exert physiological effects [162]. Maternal T4 can be transferred directly across the placental barrier and placental expression of deiodinase converts T4 to T3 [163]. When fetal thyroid hypertrophy is present, fetal thyroid dysfunction should be taken into account. Fetal thyroid disorders are mainly characterized by acquired or congenital hyperthyroidism and hypothyroidism [164]. Since hypothyroidism is much more common, it can result in severe developmental delays and mental problems, as well as permanent injury to the growing fetus’s nervous system [163].

EDCs often accumulate in the HPT axis. Human trophoblast-derived cell lines can recognize thyroid hormone transport protein transthyretin (TTR) loaded with T4 through specific cell surface receptors (high-density lipoprotein receptor scavenger receptor class B member 1 (SRB1)). EDCs have a higher affinity for TTR, resulting in insufficient T4 loading and reduced placental transfer. Particularly in early pregnancy, thyroid hormone uptake is insufficient in fetuses exposed to maternal EDCs [165]. A weighted quantile sum (WQS) regression was utilized in a Boston, Massachusetts cohort study to assess the cumulative impact of six PFAS exposures (PFOS, PFHxS, PFNA, PFOA, PFHxS, MeFOSAA, EtFOSAA, FOSA) with each thyroid hormone. The results showed a significant relationship between increased PFAS and decreased T4 levels in newborns. Especially in male newborns, PFHxS and MeFOSAA contributed most to the overall admixture hazard in the WQS regression [166]. In 44 Korean subjects, total T4 concentrations in cord blood were significantly inversely associated with concentrations of PFOS, perfluorotridecanoic acid (PFTrDA), and perfluorinated compounds (PFCs). PFCs and PFOS concentrations were likewise negatively connected with fetal T3 concentrations, while maternal serum PFTrDA concentrations were negatively correlated with cord serum T3 and T4 concentrations [167]. The results of a cohort study from Shandong, China, showed that PFAS (especially PFHxS and PFBS) were negatively correlated with THs (especially free T4 and TSH) and thyroid hormone was positively correlated with developmental quotient (DQ) in all infants, which was more significant in male infants [168]. Regardless of the infant’s sex, Guo’s study indicated that elevated levels of PFAS mixtures in cord serum correlated with higher TT4 and FT4 levels and lower TSH levels in newborns [169]. In order to calculate the relationship between maternal PFAS concentrations and TH amounts in cord serum, Hong’s research used linear regression and BKMR models. Exposure to PFOA, PFDA, and PFUdA during pregnancy was linked to higher T3 concentrations, but exposure to PFNA during pregnancy was linked to lower TSH levels and PFOA to higher levels. There were gender differences in this association. PFOA and PFNA were more highly correlated with T3/FT3 in male newborns, but PFOA and PFNA were more strongly correlated with TSH in female newborns [170]. Higher prenatal PFOS, PFHxS, and PFOA levels were associated with decreased T4 concentrations in male newborns, according to a prospective study from Boston, USA [120]. A prospective cohort from Wuhan, China, also showed that PFBS and 8:2 Cl-PFESA in cord blood serum were negatively correlated with TSH in male newborns [171].

**Figure 2:**
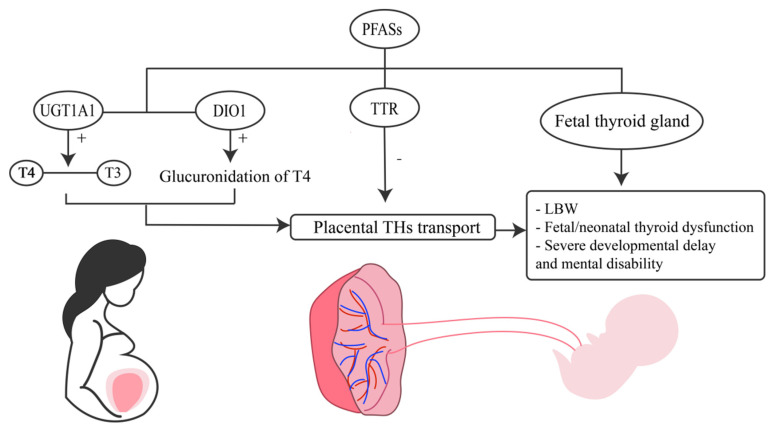
**PFAS lead to adverse pregnancy outcomes by affecting thyroid hormones.** UGT1A1: Uridine diphosphoglucuronosyl transferases, DIO1: Type 1 deiodinase, TTR: Transthyretin, THs: thyroid hormones, LBW: low birth weight.

The mechanism by which PFAS disrupts thyroid function is unknown. As an EDC, PFAS can competitively bind to TTR, and this competitive ability may result in decreased placental thyroid hormone transport [172]. PFAS may also be a thyroid hormone metabolizer. By boosting the glucuronidation of T4 by the liver enzyme uridine diphosphoglucuronosyl transferases 1A1 (UGT1A1), PFAS may improve the conversion of T4 to T3 by DIO1 [126]. In addition, PFAS have been shown in vitro to cause insufficient raw material for THs synthesis, possibly by inhibiting sodium/iodide symporter (NIS)-mediated iodine uptake by thyroid follicular cells [129]. Additionally, recent research has shown that PFAS can impair thyroid function by reducing DNA methylation [173]. However, these toxicological mechanisms do not fully explain all results, as a positive correlation between cord blood PFAS concentrations and T4 concentrations could be observed in some cohort studies, possibly because there are different toxicological effects of single PFAS [169] (Figure 2).

In some studies, it was possible to observe a sex-specific effect of PFAS on THs. PFAS, particularly PFHxS, PFOS, and PFOA, may have activating effects on estrogen receptors [174]. T4 levels rise as a result of estrogen-induced increases in thyroxine-binding globulin (TBG) levels [175]. In addition, this may explain the more significant impact of PFAS in male newborns.

### 8.5. Neurodevelopmental Disorder

Many EDCs exhibit neurotoxic properties. EDCs can be detected across various human fetal development matrices, including amniotic fluid, the placenta, and maternal blood. During early development, EDCs in the fetal environment may impair neurodevelopment, potentially leading to behavioral and neurological disorders in adulthood [176]. Research indicates that perfluoroalkyl substances (PFAS) can traverse the blood–brain and blood–cerebrospinal fluid barriers, accumulating in the brain and cerebrospinal fluid of both humans and animals [177]. However, compared to other organs such as the liver, the fetal neurological system accumulates the least amount of PFAS [178]. Children represent the primary demographic for the onset of neurodevelopmental disorders, characterized by significant functional and behavioral problems. These include movement abnormalities such as developmental coordination disorders and tic disorders, autism spectrum disorder (ASD), attention deficit/hyperactivity disorder (ADHD), intellectual disability, and communication and particular learning disorders [179]. Luo et al. assessed the neurodevelopment of two-year-old children using the Bayley Scales of Infant and Toddler Development—Third Edition (BSID-III). The findings indicated a negative association between PFNA exposure during early pregnancy and motor, cognitive, and language development in two-year-old children. The development of the language and brain was significantly impacted by exposure to PFAS combinations [180]. Wu et al. found that prenatal exposure to PFOA adversely impacts the cognitive development of young children [181]. Varsi’s study also showed an association between PFAS concentrations and infant gross motor development, suggesting that high PFAS concentrations are a risk factor for infant cognitive dysfunction [182]. However, several studies have not discovered a significant connection between cognitive performance and exposure to PFAS during pregnancy in children [183].

A Norwegian cohort study revealed that children exposed to moderate prenatal concentrations of PFOA (0.37–1.51 ng/mL) had increased odds of developing ADHD and ASD. Conversely, an inverse relationship was observed between PFUnDA, PFDA, and PFOS exposure and the odds of ADHD and/or ASD development [184]. Shin et al. also reported that prenatal exposure to PFOS and PFHxS was associated with an increased incidence of ASD in children [22]. Kim’s research indicated that children exposed to lower doses of PFAS at 2 years of age exhibited a higher likelihood of ADHD symptoms by age 8 [185]. However, a cohort study from Hokkaido, conducted by Sachiko Itoh et al., found a negative association between maternal serum PFAS levels during pregnancy and ADHD symptoms in 8-year-old children [186]. Lien et al. [187], similarly, did not identify a negative correlation between higher prenatal levels of PFNA and ADHD symptoms in 7-year-old Asian children. Furthermore, no statistically significant associations were found between PFOA, PFOS, or PFUA exposure and ADHD symptoms in the study population. Additionally, some studies have reported no impact of prenatal PFAS exposure on the risk of ADHD or ASD in children [188,189,190,191,192,193].

Evidence for an association between prenatal PFAS and IQ in children remains inconsistent. Wang et al. [194] confirmed no significant affiliation between prenatal publicity to PFAS combos and IQ in young people. This conclusion is identical to that of Lyall et al. [193]. However, Goodman’s findings showed that doubling of serum PFOA, PFOS, and PFHxS during the first trimester was inversely linked with performance IQ (PIQ) scores in male children [195]. A cohort study of mothers and infants in Taiwan found a negative relationship between prenatal PFUDA exposure and PIQ scores in 5-year-olds, whereas prenatal PFNA exposure was negatively associated with verbal IQ (VIQ) scores in 8-year-olds [196].

There may be two mechanisms by which PFAS produce neurotoxicity. First, PFAS can augment extracellular calcium influx via L-type voltage-gated calcium channels (L-VGCCs) on neuronal cell membranes or enhance calcium ion release from mitochondria and endoplasmic reticulum storage, mediated by ryanodine receptors (RyRs) and inositol 1,4,5-triphosphate receptors (IP3Rs). This abnormal increase in calcium disrupts neuronal structure and function, impairing growth, synaptogenesis, and cognitive capabilities essential for learning and memory [197]. Liu et al. found that exposure to perfluorooctanoic acid (PFOA) and perfluorooctanesulfonic acid (PFOS) significantly elevates intracellular calcium levels in hippocampal neurons, potentially leading to oxidative stress through excessive ROS production, thereby damaging neurons [198]. PFAS can also alter levels of neurotransmitters. In vitro experiments have shown that exposure to PFOA affects dopaminergic neuron (DN) differentiation. In the neuronal precursor phase (DP2), decreased levels of Tyrosine Hydroxylase (TH), a marker of dopaminergic neuron maturation, and Neurofilament Heavy (NFH) could be observed. The mature dopaminergic differentiation phase (DP3), which is a crucial protein regulating presynaptic dopamine reuptake, shows a reduction in the Dopamine Transporter (DAT) [199]. Additionally, PFOS exposure has been associated with increased levels of glutamate and apoptosis-related proteins in the hippocampus, resulting in neuronal apoptosis and compromised learning and memory capabilities [200].

## 9. Future Perspectives

The escalating global exposure to chemical pollutants has raised significant concerns regarding their impact on reproductive health in recent years. As a ubiquitous chemical, PFAS can be detected in water sources, soil, air dust, and even in a wide variety of everyday consumer goods. It is imperative to prioritize the study of PFAS due to their adverse effects on women’s reproductive health, potentially impacting infants and children.

While certain studies have identified PFAS as a risk to women’s reproductive health, others have not established a significant link. The association between PFAS exposure and preterm birth, miscarriage, and fetal growth and development remains a topic of debate. Variations in toxicokinetic parameters among different PFAS compounds may account for these discrepancies. Specifically, the distribution and elimination half-lives of PFAS vary between mammals and humans, as indicated in reference [201]. Additionally, the functional groups of PFAS, the length of the perfluoroalkyl chains, and the exposure dose are factors that influence PFAS bioaccumulation [202]. Other potential determinants of PFAS susceptibility include occupational and ethnic differences, psychosocial factors, and breastfeeding history [203,204]. Therefore, diverse regional cohort studies may yield varying outcomes, highlighting the necessity for further large-scale studies on the correlation between PFAS exposure and female reproductive health.

Gender-specific differences in prenatal PFAS accumulation have been observed in studies examining the link between PFAS exposure and adverse pregnancy outcomes, potentially due to gender dimorphism in placental physiological functions. During the 22–24 weeks of gestation, female fetuses exhibited higher placental blood flow resistance and umbilical artery pulsatility index (UA-PI) compared to male fetuses [205]. Moreover, it has been demonstrated that while the placenta of male fetuses primarily supports fetal growth and development, it is more vulnerable to the maternal environment, whereas the female fetal placenta responds to an unfavorable maternal environment, placental gene expression, and immune function change, thereby slowing fetal growth and development and ensuring survival [206]. Consequently, male fetuses in utero with heightened PFAS exposure levels seem to face higher risks of adverse pregnancy outcomes. However, there is debate concerning the relationship between gender and unfavorable pregnancy outcomes like preterm birth, miscarriage, and fetal growth limitation. Further research is warranted to explore the link between gender and adverse pregnancy outcomes like preterm birth, miscarriage, and fetal growth restriction, along with investigating the underlying factors contributing to this phenomenon.

In conclusion, this review summarizes the impact of PFAS exposure on female reproductive health, which has some implications for global public health. Although traditional PFAS are gradually banned or replaced, emerging PFAS also exhibit reproductive toxicity. Therefore, there is a pressing need to expedite the discovery of safer alternatives to emerging PFAS. Future research should focus on investigating the specific mechanisms by which PFAS affect the reproductive system in order to prevent and treat the adverse effects of PFAS on female reproductive health.

## Figures and Tables

**Table 1:** Potential effects of major legacy or emerging perfluorinated and polyfluorinated alkyl substances on female reproductive health.

Chemical Name	Geographical Origin	Design	Studied Population	Measured or Estimated PFAS Concentrations	Potential Effects	95% CI
Legacy PFAS						
PFBS	China	Case–control study	157 surgically confirmed endometriosis cases and 178 controls seeking infertility treatment because of male reproductive dysfunction	Plasma concentrations [median (IQR)] Cases 0.091 (0.088, 0.097) ng/mL, Controls 0.089 (0.085, 0.095) ng/mL	endometriosis-related infertility [18]	3.74 (2.04, 6.84)
	Shanghai, China	Nested case–control study	165 GDM cases and 330 paired controls	Maternal serum concentrations [median (IQR)] Cases 0.17 (0.09, 0.26) ng/mL, Controls 0.13 (0.07, 0.024) ng/mL	GDM [19]	2.02 (1.04, 3.79)
	Guangxi, China	Birth cohort study	122 PTB pregnant women and 1221 normal pregnant omen	Maternal serum concentrations in all study population [median (IQR)] 1.260 (0.375, 3.806) ng/mL	Preterm birth [20]	1.666 (1.033, 2.686)
PFHxS	Nanjing, China	Case–control study	120 Chinese women with overt POI and 120 healthy control subjects	Plasma concentrations [median (IQR)] Cases 0.38 (0.29, 0.67) ng/mL, Controls 0.29 (0.22, 0.37) ng/mL	POI [12]	6.63 (3.22, 13.65)
	Canadian	Longitudinal Canadian pregnancy cohort study	127 developed gestational hypertension (without preeclampsia), 49 developed preeclampsia, and 1563 normotensive control women	Maternal plasma concentrations [median (IQR)] Gestational hypertension 1.1 (0.8, 1.8) ng/mL, Preeclampsia 1.5 (1.0, 2.0) ng/mL, Normotensive 1.0 (0.7, 1.6) ng/mL	Preeclampsia [21]	1.32 (1.03, 1.70)
	California	Population-based nested case–control study of children born	239 children diagnosed with ASD and 214 general population controls	Prenatal serum concentrations [median (5th–95th)] ASD 0.50 (0.20, 1.63) ng/mL, Controls 0.40 (0.12, 1.18) ng/mL	ASD [22]	1.95 (1.02, 3.72)
PFHpS	Danish	Case–control study nested within the birth cohort	220 cases ending in miscarriage during weeks 12–22 and 220 controls ending in singleton live births	Maternal plasma concentrations [median (IQR)] Cases 0.39 (0.29, 0.49) ng/mL, Controls 0.36 (0.28, 0.45) ng/mL	Miscarriage [23]	1.8 (1.0, 3.2)
PFHpA	Guangxi, China	Birth cohort study	122 PTB pregnant women and 1221 normal pregnant women	Maternal serum concentrations in all study population [median (IQR)] 1.412 (0.470, 2.945) ng/mL	Preterm birth [20]	1.338 (1.047, 1.709)
PFOS	Nanjing, China	Case–control study	120 Chinese women with overt POI and 120 healthy control subjects	Plasma concentrations [median (IQR)] Cases 8.18 (5.50, 13.51) ng/mL, Controls 6.02 (4.24, 9.11) ng/mL	POI [12]	2.81 (1.46, 5.41)
	U.S.	Cross-sectional study (National Health and Nutrition Examination Survey)	54 women with endometriosis and 699 women without endometriosis	serum concentrations [Geometric Mean (95%CI)] Cases 16.28 (14.09, 18.81) ng/mL, Controls 13.36 (12,18. 14.66) ng/mL	Endometriosis [24]	16.28 (14.09, 18.81)
	U.S.	Cross-sectional study (National Health and Nutrition Examination Survey)	11 women with ovarian cancer and 6641 healthy control subjects	Serum concentrations in all study population [median (IQR)] 11.40 (6.45, 19.68) ng/mL	Ovarian cancer [25]	1.011 (1.011, 1.011)
	Swedish	Pregnancy cohort study	64 pregnant women developed preeclampsia and 1709 normal pregnant women	Serum concentrations in all study population [median (IQR)] 5.39 (3.95, 7.61) ng/mL	Preeclampsia [26]	2.68 (1.17, 6.12)
	Guangxi, China	Birth cohort study	122 PTB pregnant women and 1221 normal pregnant women	Maternal serum concentrations in all study population [median (IQR)] 0.892 (0.488, 1.392) ng/mL	Preterm birth [20]	1.831 (1.116, 3.005)
	Denmark	Prospective birth cohort study	112 PTB pregnant women and 3410 normal pregnant women	No concentrations are provided.	Preterm birth [27]	1.9 (1.0, 3.5)
	Ohio and West Virginia	Prospective cohort study	304 miscarriages and 1134 live births	Maternal serum concentrations [Geometric Mean] Miscarriages cases 15.0 ng/mL Controls 14.3 ng/mL	Miscarriage [28]	1.34 (1.02, 1.76)
PFOA	California	Population-based nested case–control study of children born	239 children diagnosed with ASD and 214 general population controls	Prenatal serum concentrations [median (5th–95th)] ASD 1.15 (0.37, 3.43) ng/mL, Controls 1.07 (0.37, 3.43) ng/mL	ASD [22]	(1.02, 3.72)
	Nanjing, China	Case–control study	120 Chinese women with overt POI and 120 healthy control subjects	Plasma concentrations [median (IQR)] Cases 11.10 (7.60, 14.45) ng/mL, Controls 8.35 (6.27, 11.31) ng/mL	POI [12]	3.80 (1.92, 7.49)
	Salt Lake City or San Francisco	Case–control study	190 women with endometriosis and 283 women without endometriosis	Serum concentrations [Geometric Mean (95%CI)] endometriosis cases 2.65 (2.44, 2.89) ng/mL Controls 2.15 (1.96, 2.35) ng/mL	Endometriosis [29]	1.89 (1.17, 3.06)
	U.S.	Cohort study (National Health and Nutrition Examination Survey)	54 women with endometriosis and 699 women without endometriosis	Serum concentrations [Geometric Mean (95%CI)] Cases 3.48 (2.95, 4.11) ng/mL, Controls 2.84 (2.59, 3.13) ng/mL	Endometriosis [24]	3.48 (2.95, 4.11)
	U.S.	Cross-sectional study (National Health and Nutrition Examination Survey)	11 women with ovarian cancer and 6641 healthy control subjects	Serum concentrations in all study population [median (IQR)] 3.20 (2.00, 4.90) ng/mL	Ovarian cancer [25]	1.02 (1.01, 1.02)
	Michigan and Texas, U.S.	Prospective cohort with longitudinal follow-up	28 pregnant women reported GDM during follow-up and 230 pregnant women without GDM	Serum concentrations [Geometric Mean (95%CI)] Cases 3.94 (3.15–4.93) ng/mL Controls 3.07 (2.83–3.12) ng/mL	GDM [30]	1.86 (1.14, 3.02)
	Denmark	Prospective birth cohort study	112 PTB pregnant women and 3410 normal pregnant women	No concentrations are provided.	Preterm birth [27]	1.9 (1.0, 3.6)
PFNA	Salt Lake City or San Francisco	Case–control study	190 women with endometriosis and 283 women without endometriosis	Serum concentrations [Geometric Mean (95%CI)] endometriosis cases 0.69 (0.63, 0.77) ng/mL Controls 0.58 (0.53, 0.63) ng/mL	Endometriosis [29]	2.20 (1.02, 4.75)
	U.S.	Cross-sectional study (National Health and Nutrition Examination Survey)	11 women with ovarian cancer and 6641 healthy control subjects	Serum concentrations in all study population [median (IQR)] 1.10 (0.74, 1.65) ng/mL	Ovarian cancer [25]	1.031 (1.030, 1.033)
	Danish	Case–control study within a population-based, prospective cohort	51 women with miscarriage and 204 women with full-term delivery	Serum concentrations [median (95%CI)] cases 1.16 (0.63, 2.46) ng/mL controls 0.68 (0.31, 1.35)	Miscarriage [31]	16.5 (7.4–36.6)
PFDA (PFDeA)	Danish	Case–control study within a population-based, prospective cohort	51 women with miscarriage and 204 women with full-term delivery	Serum concentrations [median (95%CI)] cases 0.33 (0.17, 0.66) ng/mL controls 0.26 (0.15, 0.56)	Abortion [31]	2.67 (1.31–5.44)
PFUnDA (PFUdA, PFUnA, PFUA)	Guangxi, China	Birth cohort study	122 PTB pregnant women and 1221 normal pregnant women	Maternal serum concentrations in all study population [median (IQR)] 0.422 (0.274, 0.660) ng/mL	Preterm birth [20]	0.621 (0.395, 0.977)
PFDOA (PFDoDA, PFDDA)	Shandong, China	Case–control study	180 infertile PCOS cases and 187 healthy controls	Plasma concentrations [median (IQR)] Cases 0.23 (0.21, 0.27) ng/mL, Controls 0.24 (0.20, 0.28) ng/mL	PCOS related infertility [32]	2.36 (1.12, 4.99)
	Shanghai, China	Nested case–control study	165 GDM cases and 330 paired controls	Maternal serum concentrations [median (IQR)] Cases 0.19 (0.04, 0.33) ng/mL, Controls 0.08 (0.02, 0.28) ng/mL	GDM [19]	2.49 (1.07, 3.72)
	Guangxi, China	Birth cohort study	145 anemia pregnant women and 676 normal pregnant women	Serum concentrations in all study population [median (IQR)] Cases 0.116 (0.084,0.158) ng/mL Controls 0.108 (0.078,0.146) ng/mL	Gestational anemia [33]	1.576 (1.107, 2.442)
Emerging PFAS						
6:2 Cl-PFESA	Shanghai, China	Case–control study	464 URSA cases who had at least 2 unexplained miscarriages and 440 normal controls	Plasma concentrations [median (IQR)] Cases 2.92 (1.41, 6.83) ng/mL, Controls 2.27 (1.00, 5.77) ng/mL	RSA [34]	1.18 (1.00, 1.39)
HFPO-DA (GENX)	Shanghai, China	Case–control study	464 URSA cases who had at least 2 unexplained miscarriages and 440 normal controls	Plasma concentrations [median (IQR)] Cases 0.03 (0.02, 0.04) ng/mL, Controls 0.03 (0.01, 0.04) ng/mL	RSA [34]	1.35 (1.15, 1.59)

Note: GDM: gestational diabetes mellitus; POI: primary ovarian insufficiency; ASD: autistic spectrum disorder; PCOS: polycystic ovary syndrome; RSA: recurrent spontaneous abortion; TSH: thyroid-stimulating hormone; FT4I: free thyroxine index.

## Abbreviations

6:2 Cl-PFESA, 6:2 Chlorinated polyfluorinated ether sulfonic acids; EtFOSAA, N-ethyl-perfluorooctane sulfonamido acetic acid; PFAS, Per- and Polyfluoroalkyl substances; PFBS, Perfluorobutane sulfonate; PFCs, Perfluorochemicals; PFCAs: Perfluorinated carboxylic acids; PFDA, Perfluorodecanoic acid; PFDoA, Perfluorododecanoic acid; PFHpA, Perfluoroheptanoic acid; PFHxS, Perfluorohexane sulfonate; PFNA, Perfluorononanoic acid; PFOA, Perfluorooctanoic acid; PFOS, Perfluoroctane sulfonate; PFSA, Perfluorosulfonic acid; PFUnDA, Perfluoroundecanoic acid; FOSA, Perfluorooctane sulfonamido acetic acid; PFHpS, Sodium Perfluoro-1-hepatanesulfonate; PFDoA, Perfluoro-n-dodecanoic acid; PFTrDA, Perfluoro-n-tridecanoic acid; PFHxPA, Perfluorohexylphosphonic acid; PFUnA, Perfluoroundecanoic acid.
